# A call for unification: how to defragment the human brain, medical professions, and national strategies?

**DOI:** 10.3325/cmj.2019.60.69

**Published:** 2019-04

**Authors:** Dinko Mitrečić, Alexandra Alves-Rodrigues, Roland Pochet

**Affiliations:** 1 Laboratory for Stem Cells, Croatian Institute for Brain Research, University of Zagreb School of Medicine, Zagreb, Croatia; 2 Croatian Brain Council, Zagreb, Croatia; 3 EU Joint Programme – Neurodegenerative Diseases Research (JPND)* dinko.mitrecic@mef.hr*; 4 Innovation Fund Denmark, Copenhagen, Denmark; 5 Université Libre de Bruxelles, Brussels, Belgium; 6 Belgian Brain Council, Brussels, Belgium

The famous quote says that if science creates problems, they will not be solved by ignorance. This is why we have to admit that the enormous amount of data produced in the last few decades, coupled to narrow specialization, brings new types of obstacles. To be an expert nowadays usually means to focus on one molecular pathway or one isolated disease, ignoring what is happening elsewhere, even if elsewhere is the laboratory next door. Clinical specialists are becoming subspecialists who pay no attention to the body regions only a few centimeters away from their field of interest. This transformation of the human body into numerous segments approached by isolated strategies represents the first level of fragmentation (1). Widening of our knowledge has become a phenomenon comparable to the expanding Universe. By making more space for the personal wisdom of our galaxies and by appearing more important to ourselves, we are becoming increasingly distant from each other. Paradoxically, measurable and accelerated widening of knowledge at the preclinical and clinical levels increases the gap between these two fields, representing the second level of fragmentation. Even worse, the expansion of our personal subfields of interest leaves less and less space for the patient, making us forget that all our professional activities exist because of the complexity of human beings affected by diseases, influencing their whole bodies and minds. There is also a third level of fragmentation – the fragmentation of education, activities, and strategies among different countries (2).

With the goal to fight fragmentation and dispersion of our knowledge and resources, we have organized the International Conference of Neurological Disorders and Neurorestoration, which will take place in Dubrovnik from May 10-13, 2019. We wanted to fill the existing gaps by organizing sessions with experts dealing with a particular group of brain diseases or strategies from various angles. For example, amyotrophic lateral sclerosis or stroke can be addressed from the point of view of neuropathology, diagnostics, or neuroinflammation; from the point view of various types of cell death; but also from the point of view of assisted ventilation in the last weeks of patient’s life (3). Every disease can and should be seen from a united point of view: a preclinical scientist is expert in molecular mechanisms and tools to control them, but does not know in what form and when clinical practitioners would require such data. And vice versa, clinicians often notice important details that should be addressed, but they barely meet scientists with whom they can design appropriate strategies to address them. The Dubrovnik conference, and this theme issue of the *Croatian Medical Journal,* offer shortcuts to bringing together various types of experts, from experts on isolated molecular pathways, to clinicians who can provide valuable feedback on offered strategies and propose new ones, and finally to patient associations voicing their interests.

Indeed, the solutions to the ever-growing burden of neurodegenerative diseases are beyond the scope and resources of any individual laboratory, group, or country alone (4). These diseases have been identified as one of the major global challenges, together with energy, climate change, and water resources. Over the last ten years, thirty countries and the European Commission have created the Joint Programming on Neurodegenerative Disease Research (JPND) – the largest global research collaboration dealing with neurodegenerative diseases. Based on a common vision, relying on approved and dynamic strategic research and innovation agenda, and supported by an agile management, JPND increases investment in transnational collaborative research among participating countries to establish causes, develop treatments, and improve the care for patients with neurodegenerative diseases and their caregivers (5). This initiative mobilizes national funding for common research actions, defragmenting and aligning national investments and research agendas. Since its first call for research proposals in 2011, JPND has supported 90 transnational research projects from EU member states and partner countries such as Canada, Switzerland, and Australia. JPND has also initiated an active partnership with US National Institute on Aging/National Institutes of Health and is particularly interested in cooperating with the countries that most recently joined the EU, also known as the EU-13 (6).

European Brain Council (EBC) is a platform that promotes and supports the cooperation between its member organizations and other stakeholders, scientific societies, patient organizations, professional societies, and industry partners. It continuously interacts with the European institutions and formulates active strategies to build strong European health policies (7). By raising awareness and encouraging education on the brain and its diseases, EBC gathers information from different disciplines to assess the repercussions of neurological and mental health conditions on society as a whole (8). Since EBC rapidly realized that its full potential can only be achieved through National Brain Councils (NBCs), it has highly supported their foundation. Considering the importance of harmonizing health policies in the large domain of brain diseases and the Croatian EU presidency starting on January 1, 2020, an integral part of the Dubrovnik conference will be the 5th NBCs Academia. A specific focus will be on better coordination and alignment, as outlined in “Ten Priorities for National Brain and Mental Health Plans,” accepted by NBCs from 17 countries.

The efforts by JPND and EBC to connect with other major transnational initiatives led to the launching of the European Brain Research Area (EBRA) Consortium in November 2018. The Consortium includes JPND, the ERANET NEURON, and the Human Brain Project, all coordinated by EBC. This brings us back to the major message: enormously growing knowledge that threatens to fragment us into numerous isolated points of interest can be overcome only by united efforts. Starting from defragmentation on national levels and harmonization of research strategies across European brain initiatives, and mirrored in more efficient alignment between preclinical and clinical levels, we will reach the needed goal – to approach the patient with unified strategies that will bring the highly needed power to disperse the black clouds of brain diseases.
